# Supplementary material for: The comparison of automated clustering algorithms for resampling representative conformer ensembles with RMSD matrix
Source: J Cheminform. 2017 Mar 23;9:21. doi: 10.1186/s13321-017-0208-0 (PMC5364127; doi:10.1186/s13321-017-0208-0)

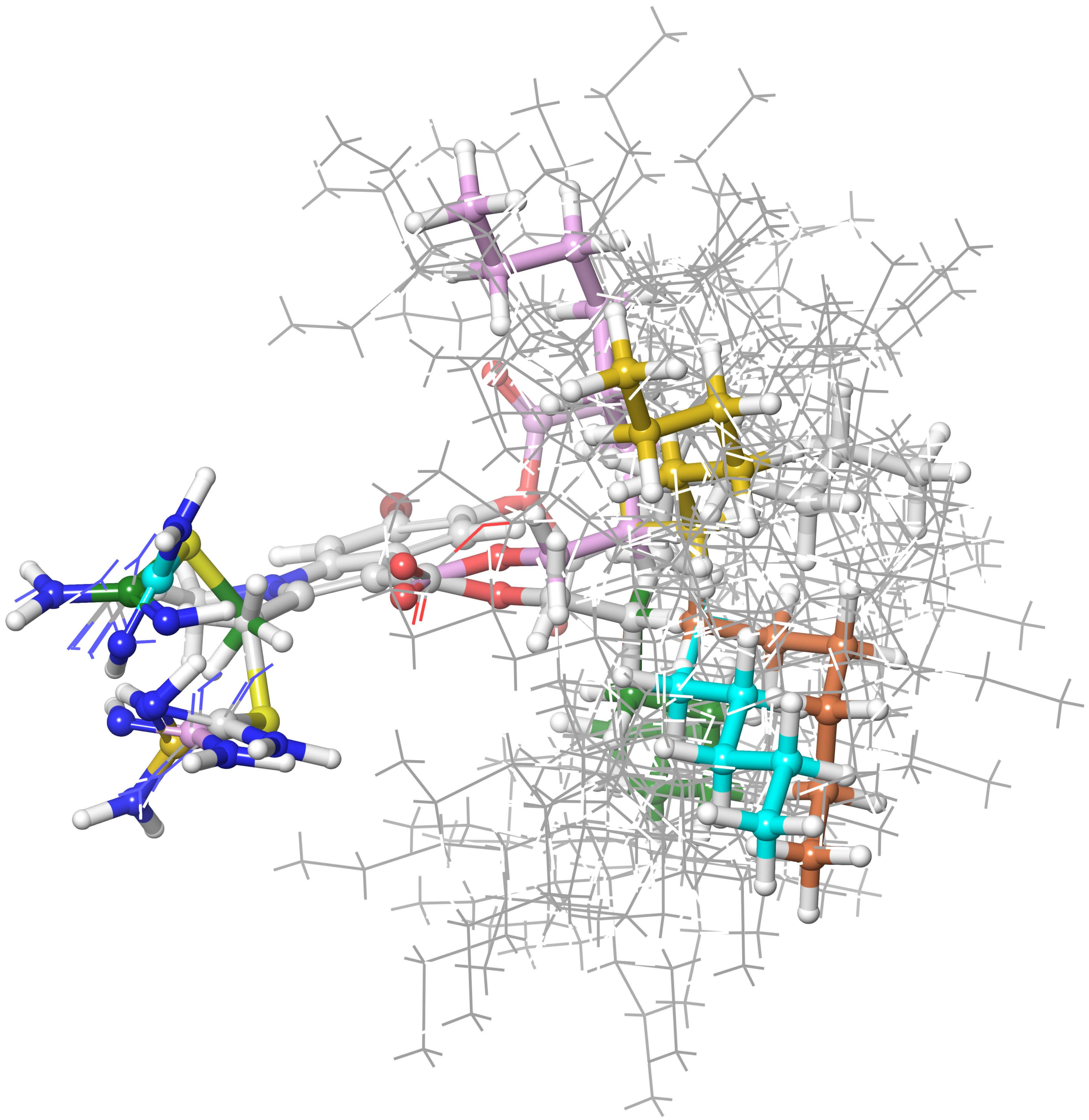

Supplement: Supplementary file 1 — Additional file 1. Conformations of every entry under RCKmeans, RCDCT, RCPCA, and RCPCA-RBF. [file 13321_2017_208_MOESM1_ESM.zip › ESM/entry_31to35.zip!/entry_34_Tree.pdf]
